# Sodium–glucose cotransporter 2 inhibitors in heart failure with preserved ejection fraction: A protocol for meta-analysis

**DOI:** 10.1097/MD.0000000000028448

**Published:** 2021-12-23

**Authors:** Hidekatsu Fukuta, Hiromi Hagiwara, Takeshi Kamiya

**Affiliations:** a Core Laboratory, Nagoya City University Graduate School of Medical Sciences, Nagoya, Japan; b Department of Medical Innovation, Nagoya City University Graduate School of Medical Sciences, Nagoya, Japan; c Department of Medical Innovation, Nagoya City University Graduate School of Medical Sciences, Nagoya, Japan.

**Keywords:** heart failure, meta-analysis, sodium–glucose cotransporter 2 inhibitors

## Abstract

**Background:**

Nearly half of patients with heart failure (HF) have preserved ejection fraction (EF) and the mortality and morbidity of patients with HF with preserved EF (HFpEF) are high. Patients with HFpEF are often elderly and their primary chronic symptom is severe exercise intolerance that results in a reduced quality of life. Thus, improvement of exercise capacity presents another important clinical outcome in HFpEF patients. Recent randomized controlled trials (RCTs) and meta-analyses of RCTs reported that sodium–glucose cotransporter 2 (SGLT-2) inhibitors improved cardiovascular outcomes in patients with HF with reduced EF. Although the effects of SGLT-2 inhibitors in HFpEF patients have been examined in multiple RCTs, the results are inconsistent due partly to limited power. The purpose of this meta-analysis is to evaluate the efficacy and safety of SGLT-2 inhibitors in HFpEF patients.

**Methods:**

This meta-analysis will include RCTs examining the effects of SGLT-2 inhibitors on HF severity and health-related quality of life in HFpEF patients. Information of studies will be collected from electronic databases. The primary outcome will be HF severity (plasma B-type natriuretic peptide levels and exercise capacity assessed as 6-minute walk distance). The secondary outcome will be health-related quality of life. The safety outcomes will be all-cause death, HF hospitalization, hypotension, acute renal failure, diabetic ketoacidosis, and urinary tract infection.

**Discussion:**

This meta-analysis will evaluate the efficacy and safety of SGLT-2 inhibitors in HFpEF patients, providing evidence to the clinical use of SGLT-2 inhibitors in these patients.

**Systematic review registration:**

INPLASY2021120033

## 1 Introduction

Nearly half of patients with heart failure (HF) in the community have preserved ejection fraction (EF) and the mortality and morbidity of patients with HF with preserved EF (HFpEF) are high.^[1–4]^ However, there is no established pharmacotherapy to improve survival in HFpEF.^[5–10]^ Patients with HFpEF are often elderly and their primary chronic symptom is severe exercise intolerance that results in a reduced quality of life.^[11,12]^ Thus, improvement of exercise capacity presents another important clinical outcome in HFpEF patients.

Recent randomized controlled trials (RCTs) and meta-analyses of RCTs reported that sodium–glucose cotransporter 2 (SGLT-2) inhibitors improved cardiovascular outcomes in patients with HF with reduced EF (HFrEF).^[13–16]^ Although the effects of SGLT-2 inhibitors in HFpEF patients have been examined in multiple RCTs,^[17–21]^ the results are inconsistent due partly to limited power.

Accordingly, the purpose of this meta-analysis is to evaluate the efficacy and safety of SGLT-2 inhibitors in HFpEF patients.

## 2 Methods

This study has been registered on International Platform of Registered Systematic Review and Meta-analysis Protocols with registration number of INPLASY2021120033 (https://www.doi.org; DOI: 10.37766/inplasy2021.12.0033). This protocol for meta-analysis will be performed according to the Preferred Reporting Items for Systematic Review and Meta-analysis Protocols statement.^[22]^

### 2.1 Search strategy

The electronic databases for literature search will include PubMed, Scopus, Cochrane Library, and Web of Science. For search of the eligible studies, the following key words and Medical Subject Heading will be used: *diastolic heart failure*, *heart failure with normal (preserved) ejection fraction*, *randomized, sodium–glucose cotransporter 2 inhibitor(s)*. Only articles published in the English language will be included.

### 2.2 Study design

RCTs will be included for this meta-analysis. Observational studies will not be included.

### 2.3 Selection criteria

Studies will be considered eligible if they: included HFpEF; were RCT; used SGLT-2 inhibitors; compared with usual medical therapy or placebo control group; and assessed HF severity.

### 2.4 Outcomes

The primary outcome will be HF severity. In the measures of HF severity, plasma B-type natriuretic peptide levels and exercise capacity assessed as 6-minute-walk distance will be extracted. The secondary outcome will be health-related quality of life assessed as the Kansas City Cardiomyopathy Questionnaire. The safety outcomes of interest will be all-cause death, HF hospitalization, hypotension, acute renal failure, diabetic ketoacidosis, and urinary tract infection.

### 2.5 Data extraction

Information on the study and patient characteristics, methodological quality, intervention strategies, and clinical outcomes will be systematically extracted separately by 2 reviewers. Disagreements will be resolved by consensus.

### 2.6 Quality assessment

The Cochrane Risk of Bias tool will be used to assess the quality of included RCTs.^[23]^ The quality of evidence for the outcomes will be evaluated by use of the Grading of Recommendations Assessment, Development and Evaluation (GRADE) system.^[24]^ The quality of evidence will be evaluated across the domains of risk of bias, consistency, directness, precision, and publication bias.

### 2.7 Statistical analysis

For continuous outcomes, the effect size for the intervention will be calculated by the difference between the means of the intervention and control groups at the end of the intervention. When available, the mean difference with corresponding standard deviation (SD), standard error of the mean (SEM), or confidence interval (CI) will be directly extracted from the article. When mean values (SD) at baseline and at the end of intervention are reported but the SD of the change or the correlation of the pre and post measurements is not available, the correlation will be conservatively set at 0.5 as previously reported.^[25]^ When the outcome is reported as median (range and/or interquartile range), the mean and SD will be estimated as previously reported.^[26]^ If the outcome is measured on the same scale, the weighted mean difference (WMD) and 95% CI will be calculated. Otherwise, the standardized mean difference and 95% CI will be calculated. For each outcome, heterogeneity will be assessed using the Cochran *Q* and *I*^2^ statistic; for the Cochran *Q* and *I*^2^ statistic, a *P* value of <.1 and *I*^2^ >50%, will be considered significant, respectively.^[27]^ When there is significant heterogeneity, the data will be pooled using a random-effects model, otherwise a fixed-effects model will be used. For categorical outcomes, the pooled estimate of odds ratio and 95% CI will be calculated with a fixed-effects model. When there is significant heterogeneity, the data will be pooled using a random-effects model. Event numbers will be either directly extracted or calculated. Publication bias will be assessed graphically using a funnel plot and mathematically using Egger test.

### 2.8 Sensitivity analysis

Meta-regression will be used to determine whether the effect of SGLT-2 inhibitors is confounded by baseline clinical characteristics. Meta-analysis will be performed separately for RCTs that included patients with EF≥50% and those that included patients with EF ≥40%.

### 2.9 Ethical issues

This meta-analysis is a literature study. Ethical approval is not required because this meta-analysis will not involve any subject directly.

## 3 Discussion

To the best of our knowledge, this is the first meta-analysis of the effect of SGLT-2 inhibitors on HF severity in HFpEF patients. The results of our meta-analysis will evaluate whether SGLT-2 inhibitors are beneficial for HFpEF patients, providing evidence regarding the clinical use of SGLT-2 inhibitors in these patients.

## Author contributions

All authors critically revised the manuscript.

**Conceptualization:** Hidekatsu Fukuta.

**Data curation:** Hiromi Hagiwara.

**Drafted manuscript:** Hidekatsu Fukuta.

**Literature retrieval:** Hidekatsu Fukuta, Hiromi Hagiwara.

**Methodology:** Hidekatsu Fukuta, Hiromi Hagiwara, Takeshi Kamiya.

**Supervision:** Takeshi Kamiya.

**Writing – original draft:** Hidekatsu Fukuta.

**Writing – review & editing:** Hiromi Hagiwara, Takeshi Kamiya.
